# Genomic incongruence accompanies the evolution of flower symmetry in Eudicots: a case study in the poppy family (Papaveraceae, Ranunculales)

**DOI:** 10.3389/fpls.2024.1340056

**Published:** 2024-06-14

**Authors:** Lisa Pokorny, Jaume Pellicer, Yannick Woudstra, Maarten J. M. Christenhusz, Teresa Garnatje, Luis Palazzesi, Matthew G. Johnson, Olivier Maurin, Elaine Françoso, Shyamali Roy, Ilia J. Leitch, Félix Forest, William J. Baker, Oriane Hidalgo

**Affiliations:** ^1^ Real Jardín Botánico (RJB-CSIC), Madrid, Spain; ^2^ Royal Botanic Gardens, Kew, Richmond, United Kingdom; ^3^ Institut Botànic de Barcelona (IBB), CSIC-CMCNB, Barcelona, Spain; ^4^ Natural History Museum of Denmark, University of Copenhagen, Copenhagen, Denmark; ^5^ Department of Environment and Agriculture, Curtin University, Perth, WA, Australia; ^6^ Jardí Botànic Marimurtra, Fundació Carl Faust, Blanes, Spain; ^7^ División Paleobotánica, Museo Argentino de Ciencias Naturales, CONICET, Buenos Aires, Argentina; ^8^ Department of Biological Sciences, Texas Tech University, Lubbock, TX, United States

**Keywords:** actinomorphy, Angiosperms353, Fumarioideae, *Hypecoum*, phylogenomics, *Pteridophyllum*, target capture sequencing, zygomorphy

## Abstract

Reconstructing evolutionary trajectories and transitions that have shaped floral diversity relies heavily on the phylogenetic framework on which traits are modelled. In this study, we focus on the angiosperm order Ranunculales, sister to all other eudicots, to unravel higher-level relationships, especially those tied to evolutionary transitions in flower symmetry within the family Papaveraceae. This family presents an astonishing array of floral diversity, with actinomorphic, disymmetric (two perpendicular symmetry axes), and zygomorphic flowers. We generated nuclear and plastid datasets using the Angiosperms353 universal probe set for target capture sequencing (of 353 single-copy nuclear ortholog genes), together with publicly available transcriptome and plastome data mined from open-access online repositories. We relied on the fossil record of the order Ranunculales to date our phylogenies and to establish a timeline of events. Our phylogenomic workflow shows that nuclear-plastid incongruence accompanies topological uncertainties in Ranunculales. A cocktail of incomplete lineage sorting, post-hybridization introgression, and extinction following rapid speciation most likely explain the observed knots in the topology. These knots coincide with major floral symmetry transitions and thus obscure the order of evolutionary events.

## Introduction

**Figure 1:**
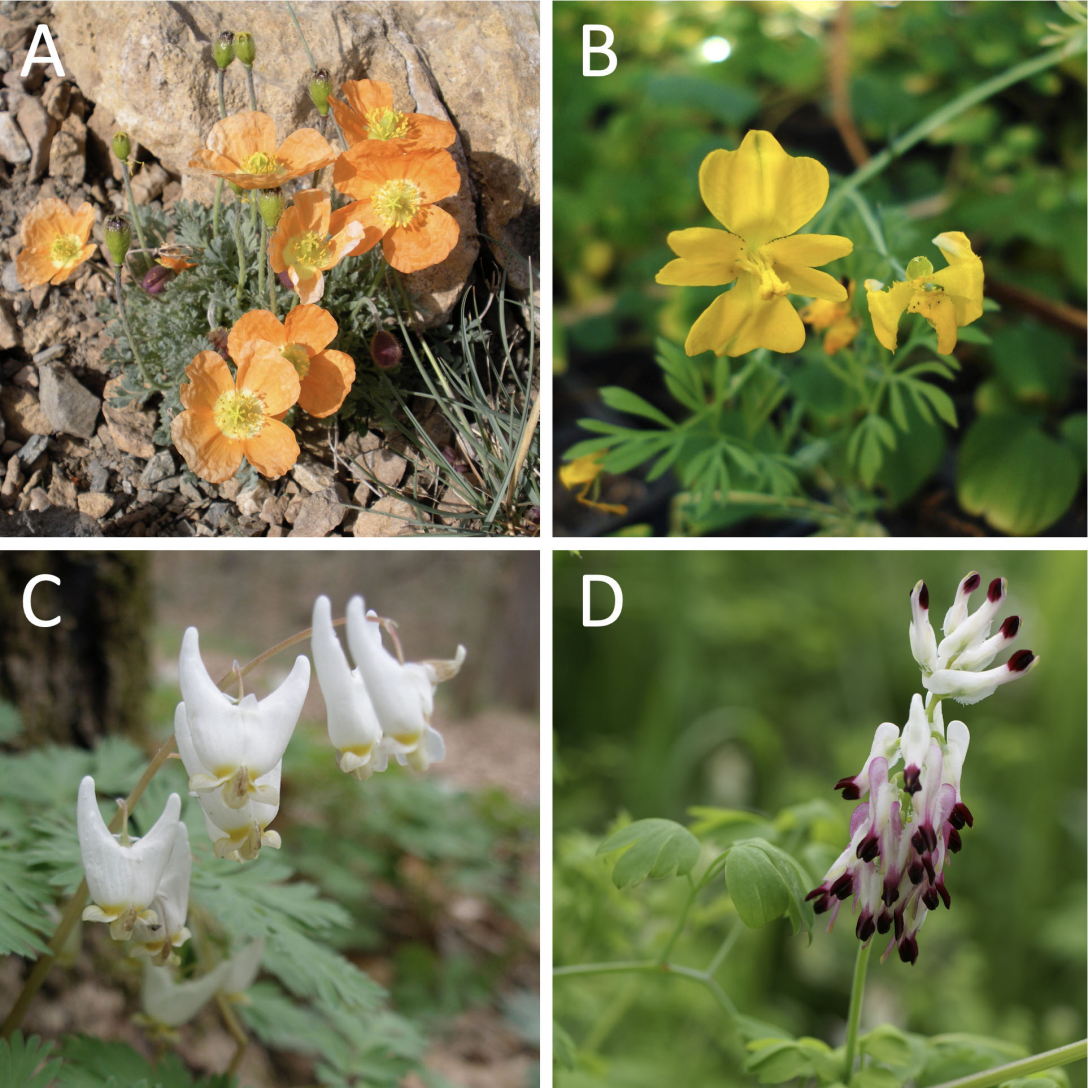
Diversity of floral symmetry in Papaveraceae. **(A)** *Oreomecon alpina* (L.) Banfi, Bartolucci, J.-M.Tison & Galasso, actinomorphic. **(B)** *Hypecoum procumbens*, disymmetric with open corolla. **(C)** *Dicentra cucullaria* (L.) Bernh., disymmetric with closed corolla. **(D)** *Fumaria capreolata* L., zygomorphic. Photograph credits: **(A)**, Pere Barnola; **(B, C)**, Oriane Hidalgo; **(D)**, Jean-Marie Martin.

The poppy family, Papaveraceae Juss. (47 genera, 1,037 species; The World Flora Online Consortium et al., 2023), belongs to the angiosperm order Ranunculales Juss. ex Bercht. & J. Presl, the sister group to all other eudicots (APG, 2016; Li et al., 2019, 2021). This key phylogenetic position, together with its astonishing floral diversity, makes the order an important model system for studying flower evolution (Damerval and Becker, 2017; Becker et al., 2023). The family Papaveraceae particularly stands out within Ranunculales and among angiosperms as a unique case of evolutionary transition in floral symmetry, from actinomorphic (radially symmetric; e.g., *Meconopsis* Vig., *Papaver* L., and *Roemeria* Medik. poppies) to disymmetric (with two perpendicular planes of symmetry; e.g., *Dicentra* Bernh. and *Lamprocapnos* Endl. bleeding hearts), and, ultimately, to zygomorphic (bilaterally symmetric; e.g., *Fumaria* L. and *Rupicapnos* Pomel fumitories) flowers (**Figure 1**; Hidalgo and Gleissberg, 2010; Sauquet et al., 2015). Disymmetry (not to be confused with dissymmetry, which means without symmetry) is rare in angiosperms (Citerne et al., 2010) and seen in Papaveraceae as an intermediate state between actinomorphy and zygomorphy (Damerval and Nadot, 2007; Sauquet et al., 2015). Additionally, molecular tools for functional validation have been developed for both actinomorphic and zygomorphic representatives, thus enabling comparative studies (Hidalgo et al., 2012; Zhao et al., 2018) and further establishing Papaveraceae as a model system for the study of floral evolution.

Papaveraceae is now widely accepted to encompass former Fumariaceae and Pteridophyllaceae families (APG, 2009). However, delimitation into subfamilies has been subject to debate in the literature, with two to four subfamilies being proposed: Fumarioideae Eaton (20 genera, including *Hypecoum* Tourn. and *Pteridophyllum* Siebold & Zucc., and 660 spp.; The World Flora Online Consortium et al., 2023) and Papaveroideae Eaton (26 genera and 377 spp.; Wang et al., 2009; Christenhusz et al., 2017) vs. Fumarioideae (excluding *Hypecoum* and *Pteridophyllum*), Hypecoideae (one genus, 18 species), Papaveroideae (as above), and monotypic Pteridophylloideae (*Pteridophyllum racemosum* Siebold & Zucc.; Hoot et al., 2015). While the phylogenetic position of *Hypecoum* as sister to the core Fumarioideae is well established (Hoot et al., 2015; Pérez-Gutiérrez et al., 2015; Sauquet et al., 2015), the placement of *Pteridophyllum* is still unclear. Indeed, the genus has been inferred as sister to all other Papaveraceae (Hoot et al., 1997, 2015), in a polytomy with Papaveroideae and Fumarioideae (Sauquet et al., 2015), and sister to Fumarioideae (incl. *Hypecoum*; Peng et al., 2023b). Wang et al. (2009) suggested close affinities between *Pteridophyllum* and *Hypecoum*, leading to them both being considered part of Fumarioideae. Nevertheless, Sauquet et al. (2015) pointed to an issue with regards to the *Pteridophyllum mat*K sequence in the aforementioned study, which explained this atypical finding. From here on, we will tentatively refer to Papaveraceae as comprising four subfamilies: Papaveroideae, Fumarioideae, Hypecoideae, and Pteridophylloideae.

The subfamilies of Papaveraceae exhibit contrasting floral morphologies (Hidalgo and Gleissberg, 2010). Papaveroideae are exclusively actinomorphic and display a trend towards polyandrous flowers (Damerval and Nadot, 2007; **Figure 1A**). Pteridophylloideae present flowers with a radially symmetric corolla and four diagonally positioned, identical stamens (De Craene and Smets, 1992; Damerval and Nadot, 2007). Flowers are disymmetric in Hypecoideae and either disymmetric or zygomorphic in Fumarioideae; each of these two subfamilies presents, however, a rather distinct floral ground plan, morphology, and floral orientation (Hidalgo and Gleissberg, 2010). The Hypecoideae flower is erect, the corolla is open, with two larger outer petals, and the androecium consists of four stamens, with two larger stamens opposite the inner petals (**Figure 1B**; Lidén, 1986; Dahl, 1989). In contrast, the disymmetric Fumarioideae flower is usually pendant (*Erhendorferia* Fukuhara & Lidén excepted), the corolla forms a closed tube, the two external petals each develop a spur, and the six-stamen androecium is arranged into two bundles (**Figure 1C**; Lidén, 1986, 1993). Disymmetry is found in the Fumarioideae genera *Lamprocapnos*, *Ehrendorferia*, *Dicentra*, *Ichtyoselmis* Lidén & Fukuhara, and *Adlumia* Raf. ex DC., which are successively sister to the remaining Fumarioideae in the most recent phylogenetic analyses, when navigating the inferred topologies from the root towards the tips (Pérez-Gutiérrez et al., 2015; Sauquet et al., 2015; Peng et al., 2023a, 2023b). Disymmetry is also observed in *Dactylicapnos* Wall., a genus whose affinities with Fumarioideae presenting zygomorphic flowers have yet to be clarified. Zygomorphy arose concurrently with the loss of one spur (Lidén, 1986, 1993) and, unusually, it develops in the transverse plane with a 90°C resupination of the pedicel resulting in a secondary vertical orientation of the flowers at anthesis (**Figure 1D**; Damerval et al., 2013). Backbone relationships supporting a floral symmetry transition to zygomorphy either place *Capnoides* Tourn. ex Adans. (zygomorphic) as the sister group to a clade comprising *Dactylicapnos* (disymmetric) and remaining zygomorphic taxa (plastid topology of Pérez-Gutiérrez et al., 2015; Sauquet et al., 2015; Peng et al., 2023a) or place *Capnoides* and *Dactylicapnos* in a poorly supported clade sister to the remaining zygomorphic Fumarioideae (nuclear topology of Pérez-Gutiérrez et al., 2015; plastome topology of Peng et al., 2023b). Both topologies lead to two possible evolutionary scenarios for symmetry transition (Sauquet et al., 2015), where zygomorphy could have evolved either once from disymmetry with a subsequent loss in *Dactylicapnos*, or twice independently in *Capnoides* and the ancestor of the remaining zygomorphic Fumarioideae.

Reconstructing evolutionary trajectories and transitions shaping floral diversity relies heavily on the phylogenetic framework on which traits are to be modelled (Soltis et al., 2003). Nowadays, phylogenetic analyses are conducted on ever-larger molecular datasets, culminating with the use of high-throughput sequencing (HTS) techniques (e.g., RNA-seq, target capture sequencing, hereafter TCS), and on more comprehensive taxonomic samplings, which benefit from the excellent performance of TCS on herbarium material (Brewer et al., 2019; Kates et al., 2021). This has meant major progress in elucidating relationships across plants and substantiating hypotheses on trait evolution, including floral characters in Ranunculales and Papaveraceae (Rasmussen et al., 2009; Hoot et al., 2015; Sauquet et al., 2015; Carrive et al., 2020; Xiang et al., 2023). However, unlike Ranunculaceae Juss. (Zhai et al., 2019; He et al., 2022), Papaveraceae have, so far, been sparsely sampled in phylogenomic reconstructions based on nuclear data (Wickett et al., 2014; One Thousand Plant Transcriptomes Initiative, 2019; Xiang et al., 2023), with the only phylogenomic studies specifically designed to address relationships within Papaveraceae based on 76 plastome protein-coding genes (Peng et al., 2023b).

In light of the above considerations, the present study aims at exploring the potential of the TCS Angiosperms353 kit (Johnson et al., 2019; Baker et al., 2021; McDonnell et al., 2021) to unravel higher-level relationships within the Ranunculales order, and more specifically those implicated in the evolutionary transition of flower symmetry in Papaveraceae.

## Materials and methods

### Taxon sampling

Sampling comprised four outgroup taxa, belonging to Sabiaceae Blume (two from *Meliosma* Blume and two from *Sabia* Colebr.) and 57 ingroup taxa (three of them duplicated for quality control purposes, them being *Capnoides*, *Euptelea* Siebold & Zucc., and *Hypecoum*) from across all Ranunculales families (**Supplementary Table S1**): seven taxa in Berberidaceae Juss., representing all three recognized subfamilies (Berberidoideae, Nandinoideae, and Podophylloideae; Hsieh et al., 2022); both monotypic Circaeasteraceae Hutch. genera (*Circaeaster agrestis* Maxim. and *Kingdonia uniflora* Balf.f. & W.W.Sm.; Sun et al., 2017, 2020); two Eupteleaceae accessions (same species, *Euptelea pleiosperma* Hook.f. & Thomson, out of two extant; Cao et al., 2016); three Lardizabalaceae R.Br., representing both broadly recognized subfamilies (two from Lardizabaloideae and one from Sargentodoxoideae; Wang et al., 2009; Christenhusz, 2012); three Menispermaceae Juss., representing both broadly agreed upon subfamilies (two from Chasmantheroideae and one from Menispermoideae; Ortiz et al., 2016); nine taxa in Ranunculaceae, representing all four recognized subfamilies (one each from Coptidoideae, Glaucidioideae, and Hydrastidoideae, and six from Ranunculoideae s.l., which includes the formerly recognized Thalictroideae; Cossard et al., 2016; Zhai et al., 2019); and, lastly, 34 taxa from Papaveraceae, representing 60% genera from all four putative subfamilies (16 Fumarioideae accessions, two Hypecoideae accessions, 15 Papaveroideae accessions, and a single Pteridophylloideae accession; Hoot et al., 2015; Sauquet et al., 2015). For each Papaveraceae subfamily, we sampled all recognized tribes (e.g., Chelidonieae, Eschscholtzieae, Papavereae, and Platystemoneae in Papaveroideae, and Corydaleae s.l. and Fumarieae in Fumarioideae).

### DNA extraction and genomic library preparation

We followed the cost-saving molecular workflow described by Hale et al. (2020) and Baker et al. (2022). Tissue samples were obtained either from silica-dried samples, fieldwork expeditions and the living collections held at RBG Kew, Graz Botanic Garden, and Utrecht University Botanic Garden, or from herbarium vouchers (Herbarium K, see also **Supplementary Table S1**). After tissue pulverization with a Mixer Mill MM400 (Retsch GmbH, Haan, Germany), DNA was extracted following a modified CTAB protocol (Doyle and Doyle, 1987), optimized for historical herbarium tissue (protocols available from Larridon et al., 2020; Shee et al., 2020), and purified with Agencourt AMPure XP magnetic beads (Beckman Coulter, Indianapolis, IN, USA). Purified DNA was quantified with a Quantus fluorometer (Promega, Madison, WI, USA), using the QuantiFluor® dsDNA Dye System, and then visualized in a 1% agarose gel to assess average fragment size distribution. When fragment sizes averaged ≥ 500 bp, purified DNA extracts were sonicated with an M220 Focused ultrasonicator, using microTUBES AFA Fiber Pre-Slit Snap-Cap (Covaris, Woburn, MA, USA), with 30–90 s shearing times (dependent on fragment size profiles) to obtain an average fragment size of ~250 bp.

Dual-indexed genomic libraries, for Illumina®, were prepared using the NEBNext® Ultra™ II DNA Library Prep Kit and the NEBNext® Multiplex Oligos (Dual Index Primers Sets 1 and 2) from New England BioLabs (Ipswich, MA, USA) at half the recommended volumes (size selection with Agencourt AMPure XP magnetic beads and eight-cycle indexing PCR). Concentration of genomic libraries was checked using the Quantus fluorometer and profiled for fragment distribution on a 4200 TapeStation System, using High Sensitivity D1000 ScreenTapes, from Agilent Technologies (Santa Clara, CA, USA). Lastly, genomic libraries were normalized (10 nM) using 10 mM Tris (pH 8.0) and pooled (~20 libraries/pool). Each pool contained ~700 ng DNA for an average fragment size of ~450 bp (including adapters and dual indexes).

### Hybridization, capture, enrichment, and sequencing

Library pools were hybridized with the Angiosperms353 v1 (Johnson et al., 2019) Daicel Arbor Biosciences myBaits Expert Predesigned Panel (Ann Arbor, MI, USA), using v4.0 chemistry for a 24 h incubation at 65°C in a Hybex Microsample Incubator (SciGene, Sunnyvale, CA, USA). Chill-out™ Liquid Wax, red (Bio-Rad, Hercules, CA, USA), was added (~30 μL) to prevent evaporation. The hybridized, biotin-labelled baits were then captured with streptavidin-coated magnetic beads, and further enriched with KAPA HiFi 2X HotStart ReadyMix PCR Kit (Roche, Basel, Switzerland), for ~12 cycles, using the i5 and i7 forward and reverse “reamp” primers described in Meyer and Kircher (2010). PCR-amplified capture pools were cleaned with Agencourt AMPure XP magnetic beads, quantified with a Quantus fluorometer, and profiled on a 4200 TapeStation System. The enriched pools were then normalized (4 nM) and multiplexed for simultaneous sequencing of up to 384 samples (Hale et al., 2020; Baker et al., 2022). Finally, multiplexed enriched pools were sequenced on an Illumina® HiSeq System producing 2 × 150 bp paired end reads at either Genewiz (Takeley, UK) or Macrogen (Seoul, South Korea).

### Data mining, sequence assembly, and refinement of data matrices

In addition to the TCS data generated, as described above, raw data from RNA-seq, whole genome sequencing (WGS), and TCS experiments (**Supplementary Table S1**) were downloaded from the NCBI Sequence Read Archive (SRA), using fastq-dump (--split-files flag) from the sra-tools package (available at https://github.com/ncbi/sra-tools). Demultiplexed reads were quality-checked with FastQC (Andrews, 2010), before and after removing adapters, low quality bases, and short reads (parameters: ILLUMINACLIP: TruSeq3-PE.fa:2:30:10 LEADING:20 TRAILING:20 SLIDINGWINDOW:4:20 MINLEN:50) with Trimmomatic v0.38 (Bolger et al., 2014).

Nuclear and plastid sequences were recovered in two separate workflows, both using HybPiper 1.3.1 (Johnson et al., 2016). These workflows take quality-filtered, paired reads and map them to either nuclear targets (mega353.fasta; McLay et al., 2021), using BWA (Li and Durbin, 2009), or to plastid targets (plastid_targets.faa; https://github.com/mossmatters/plastidTargets), using BLASTx (Altschul et al., 1990). To generate the plastid target file, a k-medoids method similar to that implemented in Johnson et al. (2019) to select representative sequences from 1KP data (https://github.com/magitz/1KP_Plastid) was used to select up to six angiosperm sequences that were within 15% sequence similarity of all 1KP angiosperm sequences for each gene. Next, these mapped, nuclear or plastid target-binned reads are assembled into *de novo* contigs with SPAdes v3.13.1 (Bankevich et al., 2012). The resulting *de novo* contigs are then refined with exonerate (Slater and Birney, 2005) and HybPiper’s intronerate.py script, keeping either nuclear exons (contigs) or plastid exons with partial, flanking introns (supercontigs). HybPiper produces summary statistics for the resulting gene data matrices relying on SAMtools (Li et al., 2009) and the hybpiper_stats.py and paralog_investigator.py scripts. Lastly, the HybPiper retrieve_sequences.py script recovers the assembled nucleotide sequences to build the corresponding nuclear contig and plastid supercontig data matrices, aided by GNU Parallel (Tange, 2018).

Additionally, the max_overlap.R script (Shee et al., 2020) was used (across accessions per gene, per genomic compartment) to compute a coverage score (for each assembled sequence) that is proportional to three statistics—representedness (proportion of accessions/genes with sequences), completeness (per cent sequence recovered against target length), and evenness (sequence length distribution across accessions/genes; Pielou, 1966)—to identify underrepresented, incomplete, and unevenly distributed sequences. For each nuclear gene contig or plastid gene supercontig, data matrices were filtered to remove genes with three or more paralogs. Following this, genes with <2/3 median coverage score values (as computed by max_overlap.R) were also discarded from downstream analyses.

### Multiple sequence alignment and filtering

Filtered data matrices were aligned with MAFFT 7.402 (Katoh and Standley, 2013), using the E-INS-i algorithm (--genafpair --maxiterate 1000), and multiple sequence alignment (MSA) summary statistics were computed with AMAS (Borowiec, 2016) to check whether, for any genes, the alignment length or the proportion of parsimony informative characters (P_PIC_) was < 1/3 median (all remaining genes passed this filter). Resulting MSAs were used to infer exploratory trees with FastTree 2 (Price et al., 2010) for automated outlier removal with TreeShrink (Mai and Mirarab, 2018), in “per-species” mode (not to confuse outgroup taxa with outliers) for various levels of false positive tolerance (α), which controls outlier detection (-q “0.05 0.5”). Pre- and post-automated outlier removal FastTree trees were visually inspected with FigTree 1.4.4 (Rambaut, 2018) to check TreeShrink performance. Outlier-filtered data matrices (0.5 threshold) were realigned (with MAFFT), and summary statistics were computed as above (none of the remaining genes had to be removed). Output MSAs were refined with trimAl (Capella-Gutierrez et al., 2009), using lax gap and conservation thresholds (-gt 0.1 -cons 35) to prevent the massive loss of data and phylogenetic signal (P_PIC_) in our patchy (albeit even) matrices, characteristic of TCS data. Once again, summary statistics were computed with AMAS (Borowiec, 2016).

### Gene and species tree inference

Nuclear gene trees were estimated for outlier-filtered, trimmed MSAs with IQ-TREE 1.5.5 (Nguyen et al., 2015) using ModelFinder Plus (Kalyaanamoorthy et al., 2017), to select the best-fit model and continue with maximum likelihood (ML) tree inference and using UFBoot, an ultrafast bootstrap approximation (Minh et al., 2013), to compute 1,000 bootstrap replicates (-m MFP -bb 1000). Resulting gene trees had bipartitions collapsed (sensitivity analysis) under various bootstrap support (BS) thresholds ('i & b<'$bs'') using the nw_ed program from the newick_utils set of programs (Junier and Zdobnov, 2010). These variously collapsed gene trees were used as input to estimate nuclear species trees with ASTRAL III 5.6.3 (Zhang et al., 2018), which was run with extensive Newick annotations (-t 2), to check whether collapsing under these various bootstrap thresholds had an effect on the resulting species tree topology and on local posterior probability (LPP) values. ASTRAL developers (Zhang et al., 2018; Mirarab, 2019) do recommend collapsing bipartitions with extremely low support, since this strategy can substantially improve accuracy. Additionally, RAxML-NG (Kozlov et al., 2019) was used to estimate branch lengths (--evaluate --brlen) in substitutions per site (rather than in coalescent units) from the species tree (inferred from gene trees with bipartitions collapsed when BS < 11) and the concatenated nuclear data matrix, generated with AMAS.

The plastome represents a canonical coalescent gene (c-gene; Doyle, 2022), and therefore, plastid outlier-filtered, trimmed supercontig alignments were concatenated into a single data matrix and partitioned by supercontig, also using AMAS, prior to phylogenomic inference. ML trees were then inferred using IQ-TREE 1.5.5 with GTR+Γ (simulations have shown that this parameter-rich model suffices in this particular case; Hoff et al., 2016; Abadi et al., 2019; Spielman, 2020) and 1,000 BS replicates (-m GTR+G -bb 1000 -bsam GENESITE), first resampling partitions and then resampling sites within them (Gadagkar et al., 2005; Seo et al., 2005).

### Divergence time estimation

Divergence times were estimated using a penalized likelihood approach as implemented in treePL (Smith and O’Meara, 2012; for a tutorial see Maurin, 2020.) In parallel, the nuclear species tree (BS < 11 bipartitions collapsed and branch lengths in substitutions per site) and the plastid ML tree (concatenated, partitioned data matrix) were used as input tree files (specifying the number of sites for nuclear and plastid alignments, respectively). After a priming run (five iterations) to determine the optimization parameters, cross-validation analyses (five iterations) were run (and optimization parameters updated as needed) to establish the smoothing parameter (smooth_NUC_ = 0.01, smooth_PL_ = 0.000001) and date the nuclear and plastid phylogenies.

Calibrations comprised minimum time constraints (from the angiosperm fossil record; compiled in Ramírez-Barahona et al., 2020) for fully supported backbone nodes, and maximum time constraints (1 Mya) for within-species tip nodes [i.e., *Euptelea pleiosperma*, *Hypecoum procumbens* L., and *Capnoides sempervirens* (L.) Borkh.]. To account for various sources of uncertainty (fossil ages, extant and extinct phylogenetic relationships, etc.), we follow Nie et al. (2020) and constrain the root using the Jurassic lower bound as a maximum time constraint (201.5 Mya), and †*Hyrcantha decussata* (Leng et Friis) Dilcher, Sun, Ji & Li and †*Tricolpites micromunus* Burger as a minimum time constraint (125 Mya; Doyle and Robbins, 1977; Dilcher et al., 2007). The crown Ranunculales node was also constrained using the Jurassic lower bound as a maximum time constraint (201.5 Mya), and the ages of †*Leefructus mirus* Sun, Dilcher, Wang & Chen, †*Potomacapnos apeleutheron* Jud & Hickey, †*Santaniella lobata* Gobo, Coiffard, Bachelier, L.Kunzmann & Iannuzzi, and †*Teixeiraea lusitanica* von Balthazar, Pedersen & Friis as a minimum time constraint (125 Mya; von Balthazar et al., 2005; Sun et al., 2011; Jud and Hickey, 2013; Vieira Gobo et al., 2022), to account for multiple sources of uncertainty (Nie et al., 2020). The crown Sabiaceae node was constrained using †*Sabia menispermoides* Knobloch & Mai as a minimum time constraint (83.4 Mya; Yang et al., 2018). For Lardizabalaceae (Wang et al., 2020), the stem node was constrained using †*Kajanthus lusitanicus* Mendes, Grimm, Pais & Friis as a minimum time constraint (110 Mya; Mendes et al., 2014), and the crown node was constrained using †*Sargentodoxa globosa* (Manchester) Manchester as a minimum time constraint (41.2 Mya; Manchester, 1994). For Menispermaceae (Jacques et al., 2011), the stem node was constrained using †*Prototinomiscium vangerowii* Knobloch & Mai as a minimum time constraint (91 Mya; Knobloch and Mai, 1986; Kadereit et al., 2019) and the crown node using †*Stephania psittaca* Jud & Gandolfo as a minimum time constraint (64.67 Mya; Jud et al., 2018). Lastly, the crown node encompassing Ranunculaceae subfamilies Coptidoideae and Ranunculoideae s.l. was constrained using †*Paleoactaea nagelii* Pigg & DeVore as a minimum time constraint (56 Mya; Pigg and DeVore, 2005).

### Character state reconstruction and data visualization and plotting

**Figure 2:**
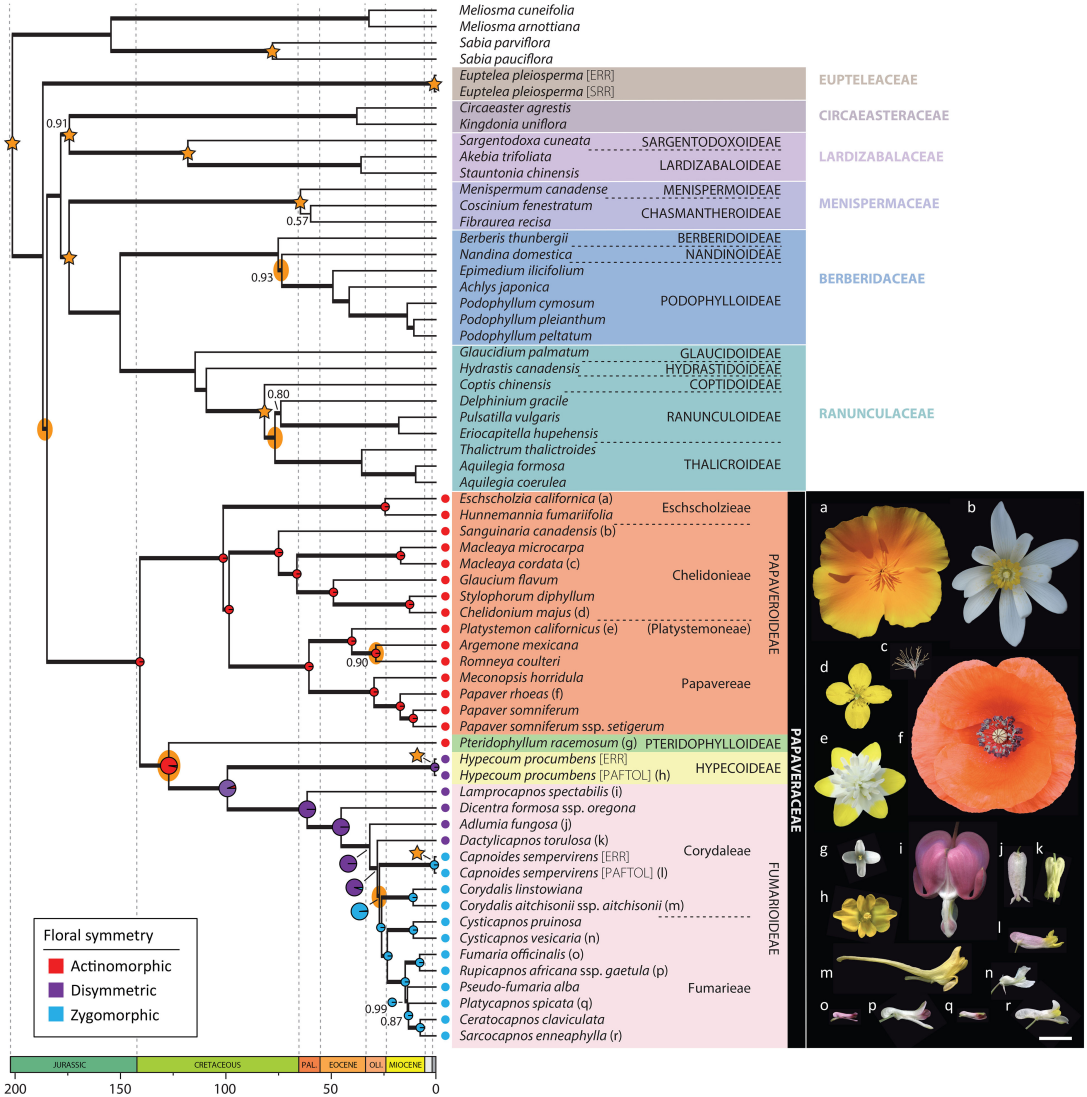
Nuclear chronogram as inferred in treePL from the ASTRAL species tree after estimating branch lengths in substitutions per site in RAxML-NG. Bold branches indicate a LPP ≥ 0.7, and support values different than LPP = 1 are provided. Stars indicate nodes with time constraints and yellow ellipses nodes with nuclear-plastid incongruence. Ancestral state reconstruction results of floral symmetry are presented in Papaveraceae as pie charts, and are larger at nodes corresponding to the transition from actinomorphy to zygomorphy. Pictures illustrate the diversity of floral morphologies in Papaveraceae, the letter next to each flower indicating the correspondence with a species of the phylogeny. Scale bar: 1 cm. Photograph credit: (a, f), Pere Barnola; (b), Oriane Hidalgo; (c, g-r), Yannick Woudstra; (d), Lisa Pokorny; (e), Maarten Christenhusz.

**Figure 3:**
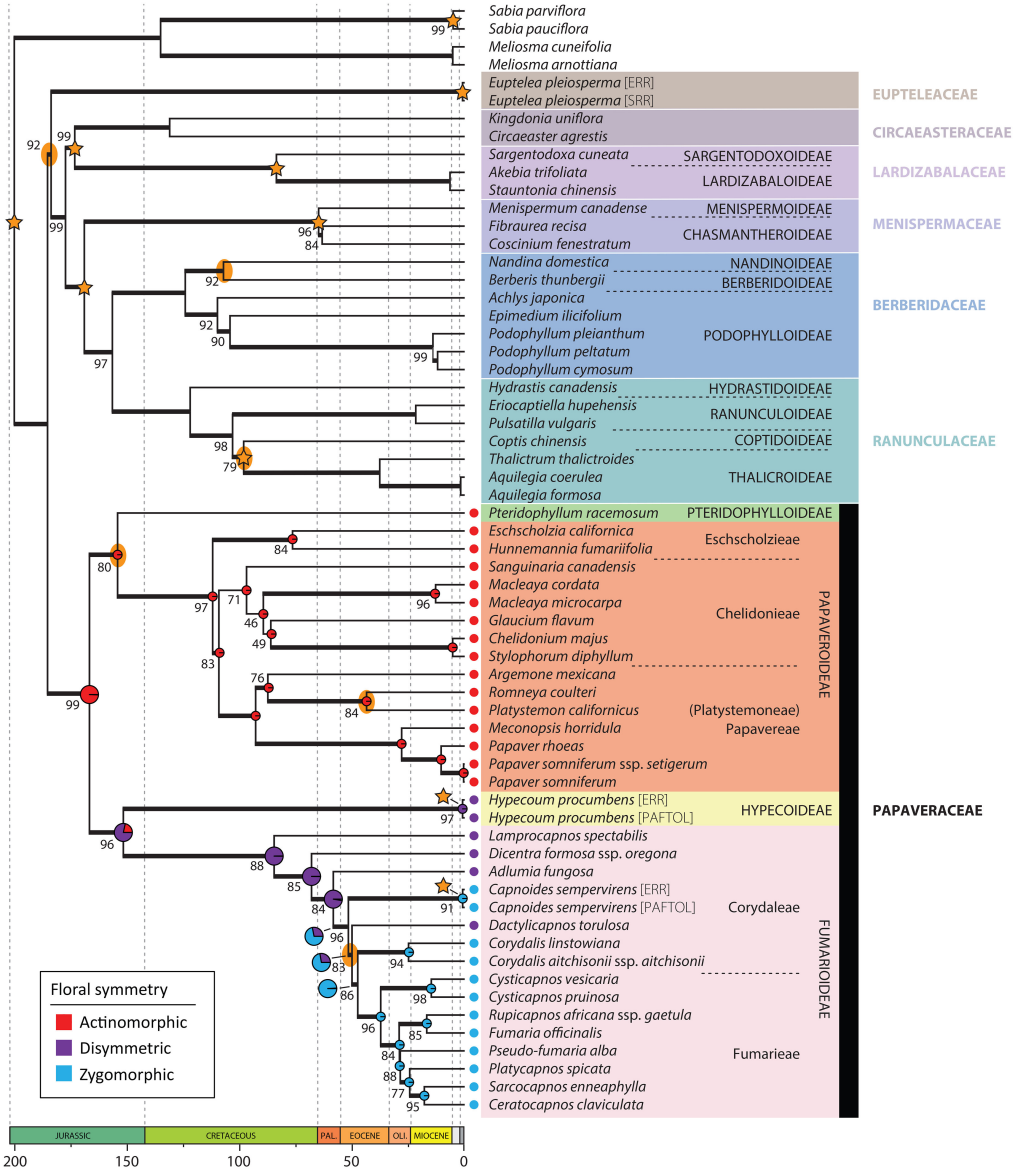
Plastid chronogram as inferred in treePL from the IQ-TREE topology estimated from the concatenated and partitioned plastid data. Bold branches indicate a BS ≥ 75%, and support values different than BS = 100% are provided. Stars indicate nodes with time constraints and yellow ellipses nodes with nuclear-plastid incongruence. Ancestral state reconstruction results of floral symmetry are presented in Papaveraceae as pie charts, larger at nodes corresponding to the transition from actinomorphy to zygomorphy.

Ancestral state reconstruction of floral symmetry was performed in R v4.2.3 (R Core Team, 2022) with RStudio v2023.03.0+386 (RStudio Team, 2022), using the ace function from the ape package (Paradis and Schliep, 2019), from within the phytools package (Revell, 2012). These analyses were performed on the nuclear and plastid chronograms, with the four Sabiaceae tips pruned. Character states for floral symmetry are indicated in **Figures 2**, **3** for Papaveraceae; remaining Ranunculales were coded as actinomorphic except for *Delphinium* L. (zygomorphic) and *Euptelea* (missing; symmetry of the perianthless *Euptelea* flower is difficult to interpret since it shifts from one to none to two axes of symmetry during development; Ren et al., 2007; Sauquet et al., 2015).

FigTree v1.4.4 (Rambaut, 2018) was used to visualize and plot most phylogenies, including the chronograms. The astralProjection function, from the AstralPlane package (Hutter, 2021), was used to plot (in R) normalized quartet support (QS) values associated with alternative quartet topologies, as inferred by ASTRAL III (Zhang et al., 2018). Also in R, functions tanglegram and untangle (step1side method), from the dendextend package (Galili, 2015), were used to visualize entanglement between the nuclear and plastid chronograms. Topologies were labelled and colored with Adobe Illustrator v26.1 (Adobe Inc, 2022).

## Results

### Target recovery and sequence and data matrix assembly

We produced *de novo* TCS data for 19 Papaveraceae, and we mined 45 NCBI SRA accessions. Three of these SRA accessions corresponded to TCS (two different probe sets), four to (shallow) WGS, and 38 to RNA-seq experiments (**Supplementary Table S1**). Following post-quality filtering with Trimmomatic, we obtained an average of 1.05M reads mapping to nuclear targets (± 1.15M SD, 0.2M min, 126.6M max), that is, an overall 17% nuclear target recovery, regardless of data provenance. From these mapped reads, a median 296 nuclear genes were recovered (with HybPiper) at 50% length (254 avg., ± 95 SD, 10 min, 337 max). For our TCS experiments, we obtained an average 2.2M reads mapping to nuclear targets (± 1.2M SD, 0.3M min, 4.9M max), out of an average 4M remaining reads post-quality filtering (± 1.8M SD, 1.5M min, 7.6M max), that is, ~50% nuclear target recovery with our cost-saving workflow (Hale et al., 2020). From these mapped reads, a median 300 nuclear genes were recovered (with HybPiper) at 50% length (296 avg., ± 29 SD, 212 min, 325 max). As for off-target plastid genes, following post-quality filtering, an average 163K reads were mapped to 72 plastid genes (± 707K SD, 41 min, 5M max), that is, an overall 12% plastid gene recovery, regardless of data provenance. From these mapped reads, a median of 44 plastid genes was recovered at 50% length (43 avg. ± 22 SD, 1 min, 71 max).

Nuclear-gene MSAs, with ≥ 2/3 median coverage score (as computed by max_overlap.R) and post paralog and outlier removal (see **Materials and methods**), had 51 taxa (± 4.7 SD, 27 min, 63 max), 650 nts length (± 764K SD, 90 min, 3.5K max), and 32K sites (± 31K SD, 4.6K min, 0.16M max), with 10.8% missing data and 0.47 P_PIC_ (median values in all instances). The concatenated nuclear data matrix comprised 64 taxa, 316 genes (out of 353), and 275,275 sites. On the other hand, resulting plastid-gene MSAs had 33 taxa (± 5.5 SD, 20 min, 42 max), 483 nts length (± 858 SD, 102 min, 3.9K max), and 15.9K sites (± 27K SD, 3K min, 0.16M max), with 6.2% missing data and 0.21 P_PIC_ (median values in all instances). The concatenated plastid data matrix comprised 62 taxa (neither *Glaucidium palmatum* Siebold & Zucc. nor *Delphinium gracile* DC. made the cut), 40 genes (out of 72), and 29,433 sites.

### Nuclear and plastid phylogenomics

Hereafter, we consider a topology as fully or maximally supported when nuclear LPP = 1.0 and plastid BS = 100%. If 1.0 > nuclear LPP ≥ 0.9 and 100% > plastid BS ≥ 95% support is high. When 0.9 > nuclear LPP ≥ 0.7 and 95% > plastid BS ≥ 75%, support is moderate. In addition, for nuclear LPP < 0.7 and plastid BS < 75%, support is weak or low. **Figure 3** shows the nuclear species tree inferred under the multispecies coalescent (MSC) from ML gene trees with bipartitions collapsed when BS < 11% (collapsing under various bootstrap thresholds had no effect on the species tree topology, but it did change topological support, **Supplementary Figure S1**). **Figure 3** shows the ML plastid phylogeny, inferred from the concatenated plastid data matrix.

**Figure 4:**
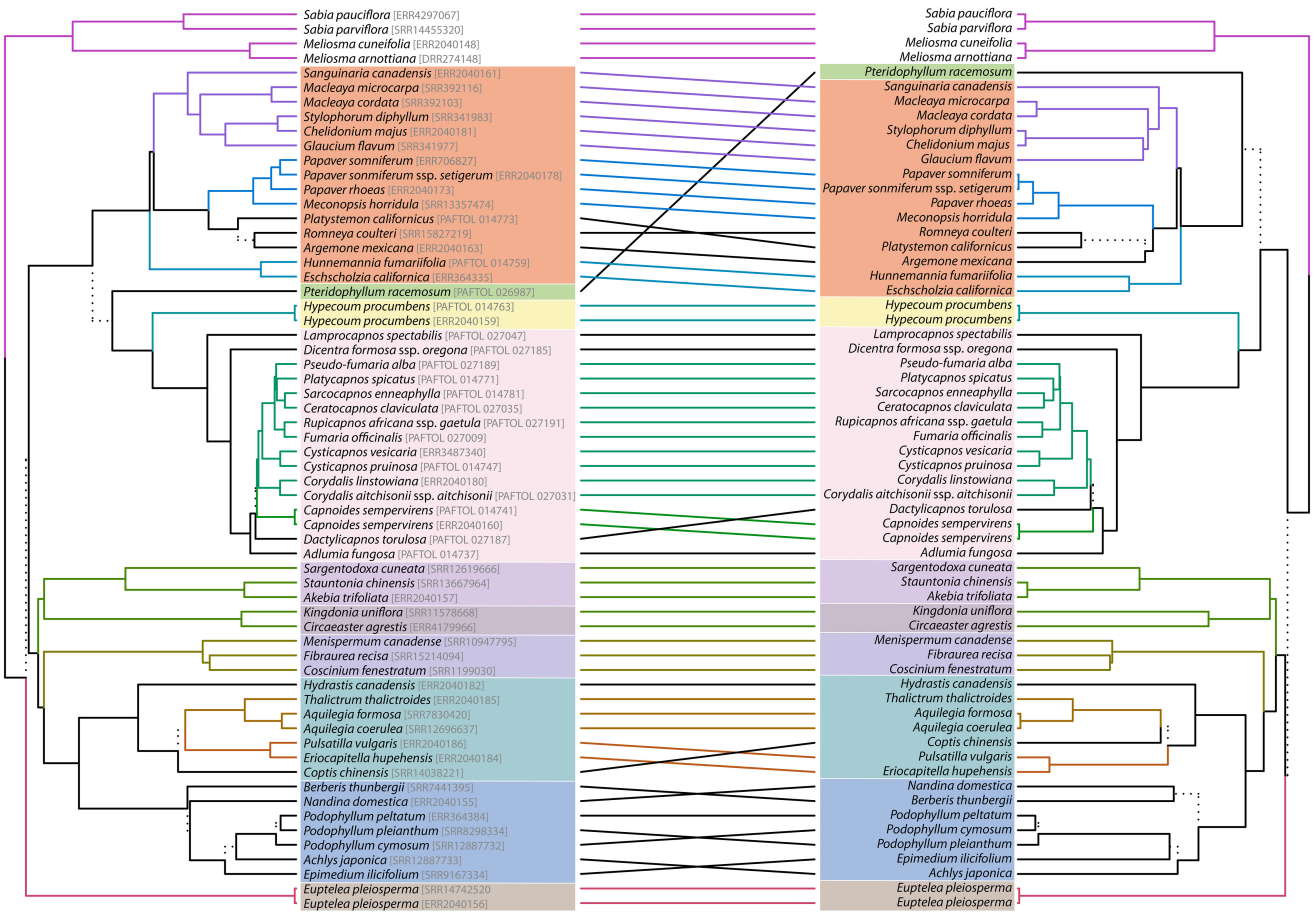
Tanglegram comparing the nuclear (left) and plastid (right) tree topology. See **Figure 3** for the correspondence of clade color. Dashed lines highlight a combination of tips just present in one topology (i.e., unique clades). Lines connecting topologies are colored to highlight subtrees present in either topology, which are also colored in the same manner.

**Figure 5:**
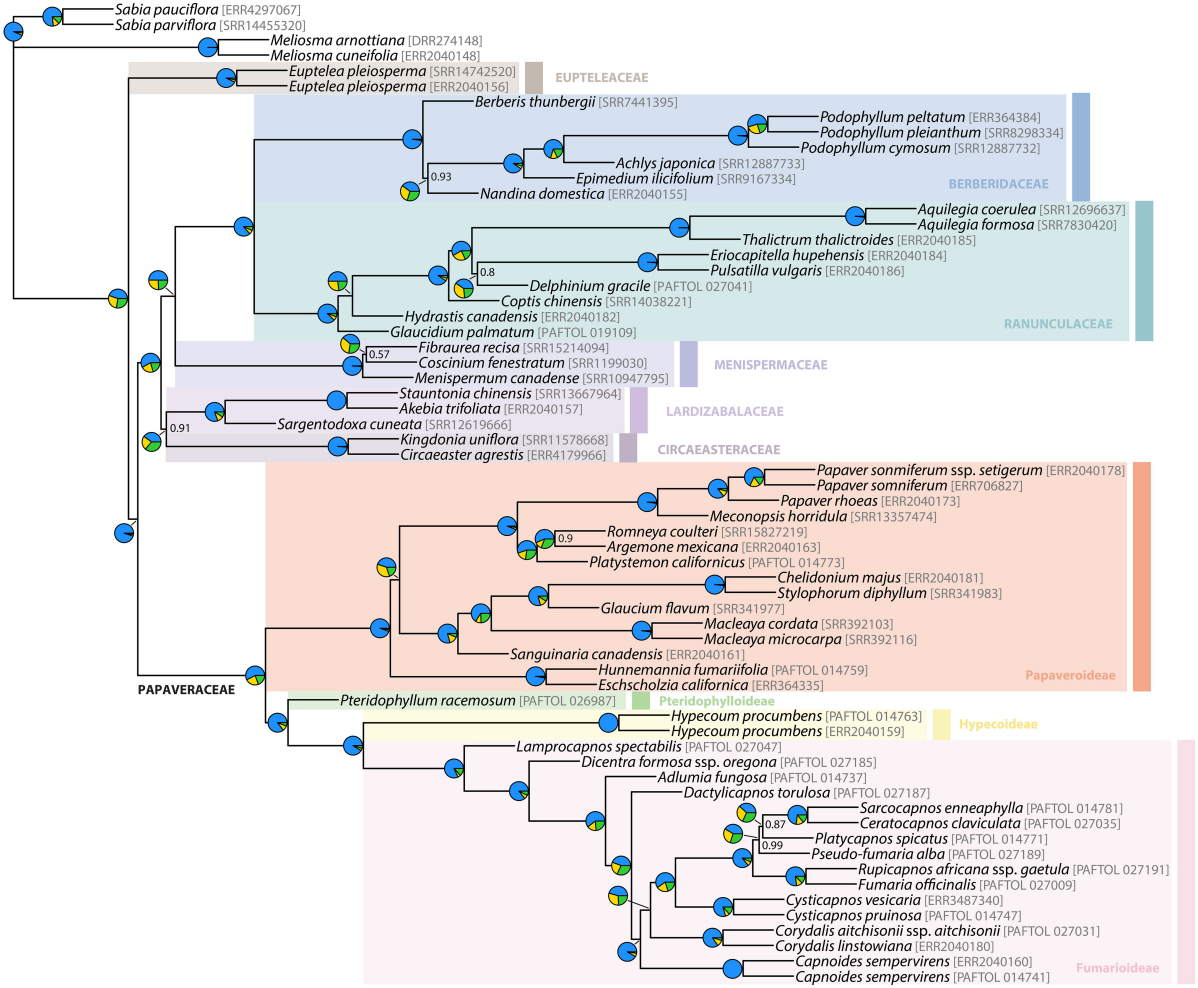
ASTRAL tree with branch lengths in coalescent units. Support values different than LPP = 1 are provided. Pies charts on branches represent normalized quartet support (QS) values for each alternative quartet topology (blue = species tree topology QS; green = first alternative topology QS; yellow = second alternative topology QS).

The nuclear topology is, for the most part, highly to maximally supported (**Figure 2**). Exceptions pertain to the weakly supported monophyly of Menispermaceae subfamily Chasmantheroideae (0.57 LPP), the moderately supported (0.8 LPP) Delphinieae plus Anemoneae clade within Ranunculoideae s.l. (Ranunculaceae), and the moderately supported placement of *Platycapnos* Bernh., as sister to *Ceratocapnos* Durieu and *Sarcocapnos* DC., within tribe Fumarieae (Fumarioideae, Papaveraceae). The plastid topology (**Figure 3**), albeit remarkably similar to the nuclear one (contentious placements discussed below), is overall more weakly supported. Specifically, the backbones of both Fumarioideae and Papaveroideae show moderate to low support throughout, with scarce highly to maximally supported placements (e.g., monophyletic genera). Both the nuclear and plastid topologies show (**Figures 2**, **3**; navigating from the tips towards the root) monophyletic Berberidaceae and Ranunculaceae families in a clade sister to a monophyletic Menispermaceae. This three-family clade is itself sister to a clade composed of monophyletic families Circaeasteraceae and Lardizabalaceae. Support is high to maximal for the above-described relationships, hereafter, Core Ranunculales clade. With regards to the placement of monophyletic families Eupteleaceae and Papaveraceae, a strong conflict is detected between our phylogenies (**Figures 4**, **5**). In the nuclear topology (**Figures 2**, **4**, **5**), Eupteleaceae is fully supported as sister to all other ranunculalean families. In the plastid tree (**Figures 3**, **4**), Papaveraceae takes that place, also with full support, while Eupteleaceae is highly supported as the sister family to the remaining ones.

Further conflict between genomic compartments can be seen within ranunculean families (**Figures 4**, **5**). In the nuclear topology (**Figures 2**, **4**, **5**), subfamily Berberidoideae (Berberidaceae) is sister to a highly supported Nandinoideae plus Podophylloideae clade; while in the plastid tree (**Figures 3**, **4**), it is Nandinoideae that is sister to a moderately supported Berberidoideae plus Podophylloideae clade. Similarly, within Ranunculaceae (navigating from the root towards the tips), a fully supported Glaucidioideae, Hydrastidoideae, and Coptidoideae grade successively leads to a Ranunculoideae clade, in the nuclear topology (**Figures 2**, **4**, **5**). By contrast, in the plastid tree (**Figures 3**, **4**), Coptidoideae is nested within Ranunculoideae s.l., with Hydrastidoideae sister to both. We could not retrieve plastid data of sufficient quality for neither Glaucidioideae nor tribe Delphinieae (Ranunculoideae s.l.) to feature in our organellar topology. Hereafter, the clade that contains subfamilies Coptidoideae and Ranunculoideae s.l. will be referred to as Core Ranunculaceae, regardless of the topology.

As for family Papaveraceae, in the nuclear topology (**Figures 2**, **4**, **5**), we infer subfamily Papaveroideae as sister to a clade where subfamily Pteridophylloideae is sister to a Hypecoideae plus Fumarioideae clade, with full support. In the plastid tree (**Figures 3**, **4**), subfamily Pteridophylloideae is instead sister to Papaveroideae, with moderate support, and this clade is in turn sister to a Hypecoideae plus Fumarioideae clade. Within Papaveroideae, tribe Platystemoneae is nested within tribe Papavereae for either topology (**Figures 2**–**4**). This broader Papavereae (including *Platystemon* Benth.) is sister to Chelidonieae, and both of them are in turn sister to Eschscholzieae, albeit with varying support for either topology. Hypecoideae and Fumarioideae are reciprocally monophyletic with high (to full) support in both phylogenies. Subfamily Fumarioideae has been further subdivided into tribes Fumarieae s.s. and Corydaleae, where the latter is paraphyletic in all our trees. With regards to the Corydaleae grade, differences between nuclear and plastid topologies pertain to the placement of *Dactylicapnos* (**Figure 4**). Navigating the fully supported nuclear topology (**Figures 2**, **5**) from root to tip, *Dactylicapnos* is sister to a clade composed of *Capnoides* and a *Corydalis* DC. plus Fumarieae s.s. clade. However, in the plastid tree (**Figure 3**), *Dactylicapnos* is sister just to a *Corydalis* plus Fumarieae s.s. clade with moderate support.

### Ranunculales chronology

As expected, divergence time estimates were similar between nuclear and plastid topologies for the nodes with time constraints, while those nodes without constraints varied (**Figures 2**, **3**). We estimate the Ranunculales crown age at 187.0_NUC_ or 185.5_PL_ Mya (Pliensbachian, Early Jurassic). For the Core Ranunculales clade, we estimate its crown age at 178.6_NUC_ or 177.5_PL_ Mya (Toarcian, Early Jurassic). Within this clade, the Menispermaceae stem node could have diverged 174.6_NUC_ or 169.1_PL_ Mya (Aalenian, Middle Jurassic), the Lardizabalaceae stem node might have split 174.4_NUC_ or 173.4_PL_ Mya (also Aalenian), the Ranunculaceae stem node could have diverged 150.3_NUC_ or 156.6_PL_ Mya (Kimmeridgian, Late Jurassic), and the Core Ranunculaceae crown node dates back to 109.4_NUC_ or 103.2_PL_ Mya (Albian, Early Cretaceous).

In the absence of fossil constraints, estimates between genomic compartments are less consistent, sometimes strikingly so, especially for sparsely sampled clades—e.g., crown Circaeasteraceae could have diverged as recently as 37.9_NUC_ Mya (Bartonian, Eocene) or as long ago as 131.2_PL_ Mya (Hauterivian, Early Cretaceous). Similarly, crown Berberidaceae might be as recent as 75.2_NUC_ Mya (Campanian, Late Cretaceous) or as old as 124.2_PL_ Mya (Barremian, Early Cretaceous). This lack of consistency can also be observed in more densely sampled clades—e.g., crown Papaveraceae could date back to 140.9_NUC_ (Berriasian, Early Cretaceous) or back to 166.8_PL_ Mya (Bathonian, Middle Jurassic); although see, crown Papaveroideae, which could have diverged 101.4_NUC_ or 112.1_PL_ Mya (Albian, Early Cretaceous). Leaving the main backbone aside, plastid divergence time estimates (**Figure 3**) are consistently older than nuclear ones (**Figure 2**).

In broad strokes, and for the nuclear chronogram (**Figure 2**), the common ancestor of Papaveraceae and Core Ranunculales could date back to the Pliensbachian. In the Early Cretaceous, in the Berriasian (140.9_NUC_ Mya), Papaveroideae and the clade composed of the remaining Papaveraceae subfamilies branch off, and in the Barremian (127.2_NUC_ Mya) Pteridophylloideae and the Hypecoideae plus Fumarioideae clade diverge. Subfamilies Hypecoideae and Fumarioideae separate in the Cenomanian (99.4_NUC_ Mya), Late Cretaceous. Within Papaveroideae, the Chelidonieae tribe crown node dates back to the Campanian (75.5_NUC_ Mya). The crowns of tribe Papavereae (including former Platystemoneae) and subfamily Fumarioideae date back to the Danian (~61 Mya). Within Fumarioideae, most of the Corydaleae grade seems to have diverged along the Oligocene (33–23 Mya), while the Fumarieae tribe crown node apparently coincides with the Paleogene/Neogene boundary (23.4_NUC_ Mya), with most of the Fumarieae s.s. diversification having taken place in the Miocene (23–5 Mya).

### Flower symmetry ancestral reconstruction

The most recent common ancestor (MRCA) of Papaveraceae is reconstructed as having actinomorphic flowers, and a single transition to disymmetric flowers is detected along the branch subtending the Hypecoideae plus Fumarioideae clade, both in the nuclear and plastid topologies (**Figures 2**, **3**). Regarding the evolution of flower symmetry in the Hypecoideae plus Fumarioideae clade, a single shift from disymmetric (not to be confused with dissymmetric, which instead means asymmetric) to zygomorphic flowers is reconstructed in the nuclear tree (**Figure 2**), whereas either two shifts to zygomorphy or one shift plus a reversal (back to disymmetry), could have taken place given the plastid topology (**Figure 3**), the latter scenario being more likely (albeit with uncertainty). In the nuclear topology (**Figure 2**), the shift from disymmetry to zygomorphy takes place along the branch subtending the clade composed of *Capnoides*, *Corydalis*, and Fumarieae s.s. In the plastid tree (**Figure 3**), *Dactylicapnos*, and not *Capnoides*, happens to be sister to a *Corydalis* plus Fumarieae s.s. clade, meaning that the transition to zygomorphy would have taken place along the branch subtending this just described clade, with a reversal along the branch leading to *Dactylicapnos*.

## Discussion

### Nuclear-plastid incongruence accompanies topological uncertainty in Ranunculales

Topological incongruence between genomic compartments in Ranunculales, as expected, centres around placements that have posed problems for a long time or that are yet to be solved (**Figures 2**, **3**, **5**), for instance, that of families Eupteleaceae and Papaveraceae, with respect to Core Ranunculales (Lane et al., 2018; Xiang et al., 2023), or the placement of genus *Pteridophyllum*, with respect to the subfamilies Papaveroideae, Hypecoideae, and Fumarioideae (see introduction). Addressing, head on, these topological incongruences and their underlying causes is key to establish a solid framework to study the evolution of flower symmetry in Eudicots.

On the one hand, in our nuclear tree (**Figures 2**, **5**) inferred from 318 putatively single-copy orthologous genes (SCOGs), Eupteleaceae is fully supported as sister to Papaveraceae and Core Ranunculales. This finding agrees with that of Lane et al. (2018) and He et al. (2022), who mined 882 and 3,611 nuclear SCOGs, respectively, from transcriptomic data and that of Xiang et al. (2023), who mined 511 low-copy nuclear genes and 42 highly conserved single-copy nuclear genes to infer relationships across Ranunculales. This topology is also recovered in the plastome analyses of Peng et al. (2023b) and Xiang et al. (2023), although with moderate support, and in the plastid phylogeny of Wang et al. (2020). On the other hand, our plastome phylogeny (**Figure 3**) moderately supports Eupteleaceae as the family sister to Core Ranunculales, rather than Papaveraceae. The organellomic approach adopted by Jin et al. (2018), which relies on whole plastome data, also places Eupteleaceae as sister to the Core Ranunculales clade with moderate support.

In our dating exercise, the MRCA of Eupteleaceae, Papaveraceae, and Core Ranunculales could have diverged as early as 185 ± 2 Mya (Pliensbachian, Early Jurassic; **Figures 2**, **3**). Wang et al. (2020) point to a slightly later divergence time (Middle Jurassic instead), while Xiang et al. (2023) infer a much later Cretaceous divergence time. We posit that the amount of (geological) time that elapsed since the Jurassic divergence we estimate from their MRCA, coupled with rapid, non-bifurcating diversification events (e.g., reticulation following polyploidy and hybridization; Morales-Briones et al., 2018, 2021, 2022) might have resulted in incomplete lineage sorting (ILS) in the nuclear compartment and might have obscured the order of relationships in the plastome, e.g., through plastid capture following hybrid speciation (Folk et al., 2016; Liu et al., 2020; Yang et al., 2021b). We argue that Eupteleaceae, which is part of the Arcto-Tertiary relict flora endemic to East Asia (Cao et al., 2016), is indeed sister to all other ranunculalean families.

### Flower symmetry transitions in Papaveraceae coincide with nuclear-plastid incongruences

Our results confirm the transition sequence of floral symmetry in Papaveraceae from an ancestor with actinomorphic flowers, to disymmetric and then zygomorphic flowers (Sauquet et al., 2015). They also highlight nuclear-plastid incongruences along with these transitions, which have different implications for reconstructing the evolutionary history of floral characters (**Figures 2**, **3**). One of these incongruences concerns *Pteridophyllum*. The genus shares close affinities with each Papaveraceae subfamily, making it difficult to determine whether the nuclear or the chloroplast topology could be the most likely one. It has actinomorphic flowers and a basal chromosome number of n = 9 otherwise found only in the Papaveroideae (e.g., in *Eomecon* Hance and *Sanguinaria* L.; Rice et al., 2015), an androecium composed of four stamens otherwise exclusive to the Hypecoideae, and a racemose inflorescence as most Fumarioideae representatives, i.e., in *Dactylicapnos* and all zygomorphic genera but for *Capnoides* (Hidalgo and Gleissberg, 2010). The nuclear inference places *Pteridophyllum* in a crucial position with its MRCA as the last representative with actinomorphic flowers before the transition to disymmetry (**Figure 2**).

The transition from disymmetry to zygomorphy (from two to a single axis of symmetry) coincides with a nuclear-plastid incongruence. The nuclear inference resulted in a novel topology (**Figure 2**), not recovered in previous studies, where *Dactylicapnos* (disymmetric flowers) is sister to a clade grouping all taxa with zygomorphic flowers, meaning a single transition from disymmetry to zygomorphy would have taken place along the stem subtending this latter zygomorphic clade. Our plastid reconstruction (**Figure 3**) results in a topology closer to previous results based on limited plastid and nuclear ribosomal data (Pérez-Gutiérrez et al., 2015; Sauquet et al., 2015; Peng et al., 2023a) and shows *Dactylicapnos* embedded within the zygomorphic clade. Flower symmetry evolution given this plastid topology would necessitate of at least two transitions: (i) one from disymmetry to zygomorphy, in the branch subtending the clade composed of all zygomorphic-flowered taxa plus *Dactylicapnos*; and (ii) a reversion to disymmetry just in *Dactylicapnos* (**Figure 3**). Increasing evidence now available on the genetic basis of floral symmetry indicates that *CYCLOIDEA*-like genes are likely involved in promoting disymmetric and zygomorphic floral development in Papaveraceae (Damerval and Nadot, 2007; Zhao et al., 2018), as is the case in most other lineages with zygomorphic flowers (Spencer and Kim, 2018). The fact that a disymmetric floral phenotype was observed in *Cysticapnos vesicaria* (L.) Fedde *CyveCYL* virus-induced gene silencing (VIGS) plants (Zhao et al., 2018) suggests a relatively simple genetic control for this floral symmetry transition. Reversion to disymmetry in *Dactylicapnos* as inferred from our plastid analyses is therefore possible (Zhang et al., 2013; Reyes et al., 2016), although the evolutionary trajectory of floral symmetry is most parsimonious in the nuclear tree.

Although the two main biological sources of nuclear-plastid conflict, i.e. hybridization and ILS, have genomic signatures that are often difficult to discriminate (Steenwyk et al., 2023; Stull et al., 2023), it is sometimes possible to differentiate between them (Morales-Briones et al., 2018, 2021, 2022; Cai et al., 2021; Meleshko et al., 2021; McLay et al., 2023). In the case of Papaveraceae, it is possible to rule out allopolyploid hybridization as a potential cause of genomic discordances at nodes corresponding to floral symmetry transitions. Analyses based on transcriptomes (including representatives of the genera *Argemone* L., *Capnoides*, *Chelidonium* L., *Corydalis*, *Cysticapnos* Mill., *Eschscholzia* Cham., *Hypecoum*, *Papaver*, and *Sanguinaria*; One Thousand Plant Transcriptomes Initiative, 2019) and on whole genome sequencing (of *Corydalis tomentella* Franch., *Eschscholzia californica* Cham., *Macleaya cordata* (Willd.) R. Br., *Papaver rhoeas* L., *P. somniferum* L., and *P. somniferum* ssp. *setigerum* (DC.) Arcang. (Yang et al., 2021a; Xu et al., 2022b) recovered no evidence of whole genome multiplication along the backbone phylogeny of Papaveraceae, from the origin of the family until after zygomorphy evolved (see **Supplementary Table 2** for a summary of ranunculean genomes sequenced to date).

### Additional considerations on the classification of Papaveraceae

The nuclear-plastid incongruence highlighted here in relation to the phylogenetic placement of *Pteridophyllum* provides further support for the consideration of the genus as constituting a subfamily of Papaveraceae in its own. Our results do not call into question the circumscription and/or phylogenetic affinities of the clades constituting the other Papaveraceae subfamilies.

At the tribe level, *Platystemon* is embedded within Papavereae in both nuclear and plastid reconstructions (**Figures 2**, **3**). This would require either including this genus (and *Hesperomecon* Greene and *Meconella* Nutt., the other constituents of Platystemoneae) in Papavereae or expanding the circumscription of Platystemoneae to include *Arctomecon* Torr. & Frém., *Argemone*, *Canbya* Parry ex A. Gray, and *Romneya* Harv. (based on our results and Peng et al., 2023b). The distribution of these genera, exclusively American, gives the group a strong biogeographical coherence (Peng et al., 2023b). However, before making a decision, it may be advisable to improve the taxonomic sampling for the nuclear data sets, so that all genera are represented (already the case in the plastid phylogeny of Peng et al., 2023b). This would also help to better delineate and understand the nuclear-plastid incongruence affecting *Romneya* (**Figures 4**, **5**), and its unusual chromosome number (2n = 38), which could result from allopolyploidization between a past or present member of the *Arctomecon* plus *Argemone* clade (2n = 28, 56, or 112 and 2n = 24, respectively) and the clade comprising *Canbya*, *Hesperomecon*, *Meconella*, and *Platystemon* (2n = 12, 14, or 16; Rice et al., 2015). Also within Papavereae, our sampling does not allow us to comment on the delimitation problems of the genera *Papaver* and *Meconopsis* highlighted by studies based on Sanger sequencing (e.g., Carolan et al., 2006; Liu et al., 2014), which would require further investigation.

In the subfamily Fumarioideae, apart from the above mentioned nuclear-plastid incongruence concerning the position of *Dactylicapnos* (**Figures 4**, **5**), our results are consistent with previous data, notably by confirming the already well-known paraphyly of the tribe Corydaleae (**Figures 3**, **4**). Reconciling classification with phylogeny would imply either that no tribe is described for this subfamily or that each genus of Corydaleae constitutes a monogeneric tribe (e.g., see Chen et al., 2023). However, to date, the only comprehensive phylogenomic framework at the genus level is based on plastid data (Peng et al., 2023b), and it would be preferable to complete the nuclear phylogenomic reconstruction before proceeding to any formal changes in the classification of the group. Given the low resolution of the nuclear inference already available (e.g., Pérez-Gutiérrez et al., 2015, based on two markers), it is difficult to anticipate the possible topological conflicts that would be thus revealed, except maybe for *Ehrendorferia*. This genus was shown to be either sister to *Dicentra* (nuclear data; Pérez-Gutiérrez et al., 2015) or isolated in a grade (plastid data; Pérez-Gutiérrez et al., 2015; Peng et al., 2023a, 2023b).

### Timing of floral symmetry and pollination syndrome shifts in Papaveraceae

Pollination in the Papaveraceae is thought to rely mostly on insects, entomophily being regarded as the ancestral mode for the family and also for the order Ranunculales (Stephens et al., 2023). The flowers of the Papaveraceae diversified from a Jurassic–Early Cretaceous ancestor with actinomorphic flowers (**Figures 2**, **3**) that most probably offered pollen as a reward, like extant representatives of the family with actinomorphic flowers. The shift to disymmetry in the Early Cretaceous (132.6–100.5 Mya, Hauterivian through Albian) coincided with important changes in floral morphology, including the formation of nectaries, which opened the way to pollination by nectar-feeding insects. However, pollination of the erect and open disymmetric flower of Hypecoideae is still partly carried out by pollen-eating insects (Dahl et al., 1990). A more specialized pollination syndrome was achieved in disymmetric Fumarioideae with a shift in flower orientation from erected to pendent (except for *Erhendorferia*), the closing of the corolla, and the formation of nectar spurs, all these characters restricting access to the reward (e.g., to long-proboscis insects; Lunau, 2004). The transition to zygomorphic flowers included the loss of a spur and the horizontal reorientation of flowers. This symmetry transition dates back to the Early Oligocene (Rupelian, 33.9–27.8 Mya), when the first Antarctic permanent ice sheets formed and a more cooling trend became established (Zachos et al., 2008). Floral symmetry changes, by enhancing pollinator specialization and thereby improving pollination efficiency, could contribute to mitigating the challenges posed by a colder climate, where resources may be limited, pollinator activity reduced, and the growing season shorter. Zygomorphy, which is considered a key innovation (i.e., a driver of diversification; O’Meara et al., 2016), is associated in Fumarioideae to yet another key innovation, the nectar spurs (Fernández-Mazuecos et al., 2019). It is then not surprising that zygomorphic Papaveraceae include this family’s most speciose genus, *Corydalis* (528 spp., representing about half of all known Papaveraceae species; The World Flora Online Consortium et al., 2023). The genus is thought to have undergone a radiation through co-evolution with insect pollinators, a subject that has received little attention in the Fumarioideae and undoubtedly merits further investigation (Damerval and Becker, 2017), beginning with the improvement of phylogenetic reconstructions for the group (Xu et al., 2022a; Peng et al., 2023a).

It can be expected that this extraordinary diversity of floral morphologies, pollination syndromes, and mating systems (Papaveraceae display a wide range of selfing rates, from primarily outcrossing to primarily selfing, meaning different degrees of dependence toward pollinators; e.g., Humphreys and Gale, 1974; Lidén, 1986; Brooks et al., 1996), which goes far beyond mere symmetry, would be reflected in pollinator networks. Unfortunately, data on pollination biology for the family are still very limited, preventing comparative analysis from being done. Nevertheless, they suggest that interactions with Diptera and Hymenoptera species predominate in Papaveroideae, whereas interactions with Hymenoptera species largely predominate in Fumarioideae (from the dataset reporting the presence of plant–pollinator species interactions in Parra et al., 2022). The rise in these insect orders in the Palaeozoic and Triassic (Asar et al., 2022) predated that of Papaveraceae; in fact, almost all Hymenoptera in these interactions are Anthophila (bees), a group that emerged in the Cretaceous (Asar et al., 2022). Our dating results are consistent with the view that Papaveraceae have co-diversified with bees and that these pollinators have likely played a major role in the increased specialization of floral phenotypes in the family, as has been suggested more generally for angiosperms (Citerne et al., 2010; Cardinal and Danforth, 2013).

The two notable exceptions from the usually entomophilous pollination of Papaveraceae are *Bocconia* Plum. ex L. and *Macleaya* R. Br., two closely related genera from South America and eastern Asia that are wind-pollinated (Li et al., 2018). Our nuclear inference dates the stem age of *Macleaya* (i.e., the split from insect-pollinated relatives; being *Bocconia* not represented in our dataset) back to the Cretaceous–Paleogene (K-Pg) boundary (**Figure 2**), close to the stem age of the *Bocconia* plus *Macleaya* clade inferred by Peng et al. (2023b). Transition to anemophily is accompanied with drastic changes in reproductive traits such as the loss of petals (**Figure 2**), the adaptation of pollen size and structure (Suárez-Santiago et al., 2018), the reallocation of reproductive resources leading to the highest pollen-to-ovule ratio in Papaveraceae (De Vos, pers. comm.), and the reorganization of the inflorescence architecture in a many-flowered diffuse panicle (Harder and Prusinkiewicz, 2013). Switching to wind pollination is seen as an almost irreversible strategy to avoid the consequences of lower pollinator activity (for example, in arid climates), when the environment is conducive to wind flow (Stephens et al., 2023).

Taken together, this study has shown the potential of the Angiosperms353 universal probe set to provide answers to lingering doubts regarding the Ranunculales backbone, evidencing that most of the uncertainty was caused by cyto-nuclear incongruence. This effort should be continued by expanding the sampling to obtain a more comprehensive phylogenomic framework onto which to model trait evolution. This would enable an in-depth study of transitions of character suites, key innovations, and co-evolution processes responsible for the extraordinary diversity of floral phenotypes this group exhibits.

## Data availability statement

The datasets presented in this study can be found in Zenodo (DOI: 10.5281/zenodo.11371203).

## Author contributions

LPo: Conceptualization, Formal analysis, Investigation, Writing – original draft. JP: Investigation, Writing – review & editing. YW: Investigation, Resources, Writing – review & editing. MC: Resources, Writing – review & editing. TG: Resources, Writing – review & editing. LPa: Writing – review & editing, Resources. MJ: Writing – review & editing, Formal analysis. OM: Writing – review & editing, Conceptualization, Data curation. EF: Investigation, Writing – review & editing. SR: Writing – review & editing, Investigation. IL: Funding acquisition, Project administration, Writing – review & editing. FF: Funding acquisition, Project administration, Writing – review & editing. WB: Funding acquisition, Project administration, Writing – review & editing. OH: Conceptualization, Formal analysis, Resources, Writing – original draft.
