# Supplementary material for: Genomic incongruence accompanies the evolution of flower symmetry in Eudicots: a case study in the poppy family (Papaveraceae, Ranunculales)
Source: Front Plant Sci. 2024 Jun 14;15:1340056. doi: 10.3389/fpls.2024.1340056 (PMC11212465; doi:10.3389/fpls.2024.1340056)

**Supplementary Table 1.** NCBI/ENA accessions mined and TCS data produced within the Plant and Fungal Trees of Life (PAFTOL) research programme at Royal Botanic Gardens, Kew (Richmond, UK). PAFTOL vouchers available at <https://treeoflife.kew.org/specimen-viewer>

| **Family** | **Subfam** | **Tribe** | **Genus** | **Taxon** | **Approach** | **NCBI/ENA** | **PAFTOL ID** |
| --- | --- | --- | --- | --- | --- | --- | --- |
| Berberidaceae | Berberidoideae | N/A | *Berberis* | *Berberis thunbergii* | RNA_seq | SRR7441395 | N/A |
| Berberidaceae | Nandinoideae | N/A | *Nandina* | *Nandina domestica* | RNA_seq | ERR2040155 | N/A |
| Berberidaceae | Podophylloideae | N/A | *Achlys* | *Achlys japonica* | RNA_seq | SRR12887733 | N/A |
| Berberidaceae | Podophylloideae | N/A | *Diphylleia* | *Diphylleia cymosa* | RNA_seq | SRR12887732 | N/A |
| Berberidaceae | Podophylloideae | N/A | *Dysosma* | *Dysosma pleiantha* | RNA_seq | SRR8298334 | N/A |
| Berberidaceae | Podophylloideae | N/A | *Epimedium* | *Epimedium ilicifolium* | RNA_seq | SRR9167334 | N/A |
| Berberidaceae | Podophylloideae | N/A | *Podophyllum* | *Podophyllum peltatum* | RNA_seq | ERR364384 | N/A |
| Circaeasteraceae | N/A | N/A | *Circaeaster* | *Circaeaster agrestis* | DNA_TCS | ERR4179966 | N/A |
| Circaeasteraceae | N/A | N/A | *Kingdonia* | *Kingdonia uniflora* | RNA_seq | SRR11578668 | N/A |
| Eupteleaceae | N/A | N/A | *Euptelea* | *Euptelea pleiosperma* | RNA_seq | ERR2040156 | N/A |
| Eupteleaceae | N/A | N/A | *Euptelea* | *Euptelea pleiosperma* | DNA_WGS | SRR14742520 | N/A |
| Lardizabalaceae | Lardizabaloideae | Lardizabaleae | *Akebia* | *Akebia trifoliata* | RNA_seq | ERR2040157 | N/A |
| Lardizabalaceae | Lardizabaloideae | Lardizabaleae | *Stauntonia* | *Stauntonia chinensis* | DNA_WGS | SRR13667964 | N/A |
| Lardizabalaceae | Sargentodoxoideae | N/A | *Sargentodoxa* | *Sargentodoxa cuneata* | DNA_WGS | SRR12619666 | N/A |
| Menispermaceae | Chasmantheroideae | Burasaieae | *Fibraurea* | *Fibraurea recisa* | RNA_seq | SRR15214094 | N/A |
| Menispermaceae | Chasmantheroideae | Coscinieae | *Coscinum* | *Coscinum fenestratum* | RNA_seq | SRR1199030 | N/A |
| Menispermaceae | Menispermoideae | Menispermeae | *Menispermum* | *Menispermum canadense* | RNA_seq | SRR10947795 | N/A |
| Papaveraceae | Fumarioideae | Corydaleae | *Adlumia* | *Adlumia fungosa* | DNA_TCS | ERR7621180 | 014737 |
| Papaveraceae | Fumarioideae | Corydaleae | *Capnoides* | *Capnoides sempervirens* | RNA_seq | ERR2040160 | N/A |
| Papaveraceae | Fumarioideae | Corydaleae | *Capnoides* | *Capnoides sempervirens* | DNA_TCS | ERR7621182 | 014741 |
| Papaveraceae | Fumarioideae | Corydaleae | *Corydalis* | *Corydalis aitchisonii* ssp. *aitchisonii* | DNA_TCS | ERR7622611 | 027031 |
| Papaveraceae | Fumarioideae | Corydaleae | *Corydalis* | *Corydalis linstowiana* | RNA_seq | ERR2040180 | N/A |
| Papaveraceae | Fumarioideae | Corydaleae | *Dactylicapnos* | *Dactylicapnos torulosa* | DNA_TCS | ERR7622618 | 027187 |
| Papaveraceae | Fumarioideae | Corydaleae | *Dicentra* | *Dicentra formosa* ssp. *oregona* | DNA_TCS | ERR7622617 | 027185 |
| Papaveraceae | Fumarioideae | Corydaleae | *Lamprocapnos* | *Lamprocapnos spectabilis* | DNA_TCS | ERR7622615 | 027047 |
| Papaveraceae | Fumarioideae | Fumarieae | *Ceratocapnos* | *Ceratocapnos claviculata* | DNA_TCS | ERR7622612 | 027035 |
| Papaveraceae | Fumarioideae | Fumarieae | *Cysticapnos* | *Cysticapnos pruinosa* | DNA_TCS | ERR7621183 | 014747 |
| Papaveraceae | Fumarioideae | Fumarieae | *Cysticapnos* | *Cysticapnos vesicaria* | RNA_seq | ERR3487340 | N/A |
| Papaveraceae | Fumarioideae | Fumarieae | *Fumaria* | *Fumaria officinalis* | DNA_TCS | ERR7622609 | 027009 |
| Papaveraceae | Fumarioideae | Fumarieae | *Platycapnos* | *Platycapnos spicata* | DNA_TCS | ERR7621186 | 014771 |
| Papaveraceae | Fumarioideae | Fumarieae | *Pseudo-fumaria* | *Pseudo-fumaria alba* | DNA_TCS | ERR7622619 | 027189 |
| Papaveraceae | Fumarioideae | Fumarieae | *Rupicapnos* | *Rupicapnos africana* ssp. *gaetula* | DNA_TCS | ERR7622620 | 027191 |
| Papaveraceae | Fumarioideae | Fumarieae | *Sarcocapnos* | *Sarcocapnos enneaphylla* | DNA_TCS | ERR7621188 | 014781 |
| Papaveraceae | Hypecoideae | Hypecoeae | *Hypecoum* | *Hypecoum procumbens* | RNA_seq | ERR2040159 | N/A |
| Papaveraceae | Hypecoideae | Hypecoeae | *Hypecoum* | *Hypecoum procumbens* | DNA_TCS | ERR7621185 | 014763 |
| Papaveraceae | Pteridophylloideae | Pteridophylleae | *Pteridophyllum* | *Pteridophyllum racemosum* | DNA_TCS | ERR7622608 | 026987 |
| Papaveraceae | Papaveroideae | Chelidonieae | *Chelidonium* | *Chelidonium majus* | RNA_seq | ERR2040181 | N/A |
| Papaveraceae | Papaveroideae | Chelidonieae | *Glaucium* | *Glaucium flavum* | RNA_seq | SRR341977 | N/A |
| Papaveraceae | Papaveroideae | Chelidonieae | *Macleaya* | *Macleaya cordata* | RNA_seq | SRR392103 | N/A |
| Papaveraceae | Papaveroideae | Chelidonieae | *Macleaya* | *Macleaya microcarpa* | RNA_seq | SRR392116 | N/A |
| Papaveraceae | Papaveroideae | Chelidonieae | *Sanguinaria* | *Sanguinaria canadensis* | RNA_seq | ERR2040161 | N/A |
| Papaveraceae | Papaveroideae | Chelidonieae | *Stylophorum* | *Stylophorum diphyllum* | RNA_seq | SRR341983 | N/A |
| Papaveraceae | Papaveroideae | Eschscholzieae | *Eschscholzia* | *Eschscholzia californica* | RNA_seq | ERR364335 | N/A |
| Papaveraceae | Papaveroideae | Eschscholzieae | *Hunnemannia* | *Hunnemannia fumariifolia* | DNA_TCS | ERR7621184 | 014759 |
| Papaveraceae | Papaveroideae | Papavereae | *Argemone* | *Argemone mexicana* | RNA_seq | ERR2040163 | N/A |
| Papaveraceae | Papaveroideae | Papavereae | *Meconopsis* | *Meconopsis horridula* | RNA_seq | SRR13357474 | N/A |
| Papaveraceae | Papaveroideae | Papavereae | *Papaver* | *Papaver rhoeas* | RNA_seq | ERR2040173 | N/A |
| Papaveraceae | Papaveroideae | Papavereae | *Papaver* | *Papaver setigerum* | RNA_seq | ERR2040178 | N/A |
| Papaveraceae | Papaveroideae | Papavereae | *Papaver* | *Papaver somniferum* | RNA_seq | ERR706827 | N/A |
| Papaveraceae | Papaveroideae | Papavereae | *Romneya* | *Romneya coulteri* | DNA_TCS | SRR15827219 | N/A |
| Papaveraceae | Papaveroideae | Platystemoneae | *Platystemon* | *Platystemon californicus* | DNA_TCS | ERR7621187 | 014773 |
| Ranunculaceae | Coptidoideae | Coptideae | *Coptis* | *Coptis chinensis* | RNA_seq | SRR14038221 | N/A |
| Ranunculaceae | Glaucidioideae | N/A | *Glaucidium* | *Glaucidium palmatum* | DNA_TCS | ERR7621844 | 019109 |
| Ranunculaceae | Hydrastidoideae | N/A | *Hydrastis* | *Hydrastis canadensis* | RNA_seq | ERR2040182 | N/A |
| Ranunculaceae | Ranunculoideae | Anemoneae | *Eriocapitella* | *Eriocapitella hupehensis* | RNA_seq | ERR2040184 | N/A |
| Ranunculaceae | Ranunculoideae | Anemoneae | *Pulsatilla* | *Pulsatilla vulgaris* | RNA_seq | ERR2040186 | N/A |
| Ranunculaceae | Ranunculoideae | Delphinieae | *Delphinium* | *Delphinium gracile* | DNA_TCS | ERR7622614 | 027041 |
| Ranunculaceae | Thalictroideae | N/A | *Aquilegia* | *Aquilegia coerulea* | RNA_seq | SRR12696637 | N/A |
| Ranunculaceae | Thalictroideae | N/A | *Aquilegia* | *Aquilegia formosa* | RNA_seq | SRR7830420 | N/A |
| Ranunculaceae | Thalictroideae | N/A | *Thalictrum* | *Thalictrum thalictroides* | RNA_seq | ERR2040185 | N/A |
| Sabiaceae | N/A | N/A | *Meliosma* | *Meliosma arnottiana* | RNA_seq | DRR274148 | N/A |
| Sabiaceae | N/A | N/A | *Meliosma* | *Meliosma cuneifolia* | RNA_seq | ERR2040148 | N/A |
| Sabiaceae | N/A | N/A | *Sabia* | *Sabia parviflora* | DNA_WGS | SRR14455320 | N/A |
| Sabiaceae | N/A | N/A | *Sabia* | *Sabia pauciflora* | DNA_TCS | ERR4297067 | N/A |

**Supplementary Table 2. Ranunculalean Whole Genomes Sequenced (NCBI & CNGB)**. FCM stands for flow cytometry, CCDB stands for Chromosome Counts Database, ORF stands for open reading frame.

| **Family (*aceae)** | **Subfamily (†oideae) / Tribe (‡eae)** | **Taxon** | **Common name** | **FCM genome size (1C in pg)** | **CCDB (2n)** | **Contigs (Hi-C scaffolds)** | **Total length (Gbp)** | **Longest contig (Mbp)** | **N50 length (Mbp)** | **GC content (%)** | **Genes / ORFs** | **NCBI / CNGB ID** | **Ref.** |
| --- | --- | --- | --- | --- | --- | --- | --- | --- | --- | --- | --- | --- | --- |
| Berberid* | Berberid† | *Berberis thunbergii* ‘Kobold’ | Japanese barberry | 1.72 (1.55§) | 28 | 2698 (14) | 1.20 | 99.76 | 88.62 | 37.66 | 55,186 | GCA_003290165 | Bartaula et al. [(2019)](https://www.zotero.org/google-docs/?vgrOvl) |
| Circaeaster* | N/A | *Kingdonia uniflora* | du ye cao | 1.15 | 18 | 2,932 | 1.00 | 11.53 | 2.09 | 38.04 | 43,301 | GCA_014058105 | Sun et al. [(2020)](https://www.zotero.org/google-docs/?ebOsPO) |
| Lardizabal* | Lardizabal† | *Akebia trifoliata* ssp. *australis* | akebi | 0.76§ | 32 | 689 (16) | 0.68 | 6.4 | 43.11 | 35.02 | 25,598 | GCA_017979445 | Huang et al. [(2021)](https://www.zotero.org/google-docs/?XOxaMu) |
| Papaver* | Fumari†/‡ | *Corydalis tomentella* | mao huang jin | 0.58 | 16 | 1,321 (8) | 0.96 | 9.83 | 2.52 | 36.79 | 37,808 | PRJCA003323 & GWHAORS00000000 | Xu et al. [(2022)](https://www.zotero.org/google-docs/?B2qE7M) |
| Papaver* | Papaver†/‡ | *Eschscholzia californica* | California poppy | 0.504 (1.10§) | 12 | 53,253 | 0.87 | 4.0 | 0.75 | 41.2 | 41,612 | GCA_002897215 | Hori et al. [(2018)](https://www.zotero.org/google-docs/?8tY4Ad), Becker et al. [(2023)](https://www.zotero.org/google-docs/?qMVrKt) |
| Papaver* | Papaver†/‡ | *Papaver armeniacum* | Armenian poppy | ? | 14 | 383,854 | 2.98 | 0.07 | 0.003 | ? | 53,981 | GCA_023531295 | Catania et al. [(2022)](https://www.zotero.org/google-docs/?05l6eI) |
| Papaver* | Papaver†/‡ | *Papaver atlanticum* | Atlas poppy | 2.30§ | 14 | 27,327 | 0.98 | 31.97 | 0.11 | ? | 33,259 | GCA_023531105 | Catania et al. [(2022)](https://www.zotero.org/google-docs/?V50MCo) |
| Papaver* | Papaver†/‡ | *Papaver bracteatum* | Persian or great scarlet poppy | 3.08§ | 14 | 223,094 | 2.15 | 2.20 | 0.02 | ? | 37,949 | GCA_023529315 | Catania et al. [(2022)](https://www.zotero.org/google-docs/?uoJwxl) |
| Papaver* | Papaver†/‡ | *Papaver californicum* | fire poppy | ? | ? | 35,487 | 1.37 | 29.34 | 0.07 | ? | 42,926 | GCA_023531435 | Catania et al. [(2022)](https://www.zotero.org/google-docs/?h0G7nq) |
| Papaver* | Papaver†/‡ | *Papaver nudicaule* | Iceland poppy | 1.80 (4.10§) | 14  (28, 42) | 353,025 | 1.59 | 0.42 | 0.06 | ? | 30,973 | GCA_023529015 | Catania et al. [(2022)](https://www.zotero.org/google-docs/?RD7z0v) |
| Papaver* | Papaver†/‡ | *Papaver rhoeas* | common poppy | 2.5 (2.41§) | 14 | 3,273 (7) | 2.5 | 39.02 | 5.29 | 37.94 | 41,470 | PRJNA720042 & GWHAZPI00000000 | Yang et al. [(2021)](https://www.zotero.org/google-docs/?lSfKw4), Zhang et al. [(2023)](https://www.zotero.org/google-docs/?bymjAw) |
| Papaver* | Papaver†/‡ | *Papaver setigerum* | Troy poppy | 4.6 (4.0§) | 44 | 553 (22) | 4.6 | 178.78 | 65.57 | 36.88 | 106,517 | PRJNA720042 & GWHAZPH00000000 | Yang et al. [(2021)](https://www.zotero.org/google-docs/?G6wXz5), Zhang et al. [(2023)](https://www.zotero.org/google-docs/?Vjtrew) |
| Papaver* | Papaver†/‡ | *Papaver somniferum* ‘HN1’ | opium poppy | 2.7 (3.8§) | 22 | 61,801 (11) | 2.7 | 13.77 | 1.74 | 37.28 | 55,316 | GCA_003573695 | Guo et al. [(2018)](https://www.zotero.org/google-docs/?1VmuwY), Li et al. [(2020)](https://www.zotero.org/google-docs/?nrSqHW), Yang et al. [(2021)](https://www.zotero.org/google-docs/?96We5f), Zhang et al. [(2023)](https://www.zotero.org/google-docs/?77S9Ga) |
| Papaver* | Papaver†/ Chelidoni‡ | *Macleaya cordata* | plume poppy | 0.54 | 20 | 4,551 | 0.38 | 5.6 | 25 | ? | 22,328 | GCA_002174775 | Liu et al. (2017) |
| Papaver* | Papaver†/ Chelidoni‡ | *Sanguinaria canadensis* | bloodroot/ red puccoon | 2.6 (1.16§) | 18 | 253,391 | 2.6 | 72.6 | 1,050 | ? | ? | GCA_028566195 | Iridian Genomes; Pirro & Pirro [(2023)](https://www.zotero.org/google-docs/?MFWE3l) |
| Ranuncul* | Coptid†/‡ | *Coptis chinensis* | goldthread | 1.15 (0.98) | 18 | 1,801 (9) | 0.937 | 4,844 | 806.6 | 38.5 | 41,004 | GCA_015680905 | Liu et al. [(2021)](https://www.zotero.org/google-docs/?FkSNfE) |
| Ranuncul* | Ranuncul†/ Isopyr‡ | *Aquilegia coerulea* ‘Goldsmith’ | Colorado blue columbine | 0.3 | 14 | 2,529 (7) | 2.988 | 6.7 | 3.1 | 37.0 | 29,550 | GCA_002738505 | Filiault et al. [(2018)](https://www.zotero.org/google-docs/?El2GDa) |
| Ranuncul* | Ranuncul†/ Isopyr‡ | *Aquilegia eximia* | Van Houtte's columbine | 0.37 | (14) | 463 | 3.67 | 18 | 4.95 | 37.6 | ? | GCA_023053565 | CCGP, UCLA / La Kretz CCCS; Johns et al. [(2022)](https://www.zotero.org/google-docs/?QInSVx) |
| Ranuncul* | Ranuncul†/ Isopyr‡ | *Aquilegia oxysepala* var. *kansuensis* | Oriental columbine | 0.312 | 14 | 852 (7) | 0.285 | 7.8 | 2.2 | ? | 25,571 | GCA_020826895 | Xie et al. [(2020)](https://www.zotero.org/google-docs/?jqupZq) |
| Ranuncul* | Ranuncul†/ Isopyr‡ | *Thalictrum thalictroides* | rue-anemone | 3.66§ | 14 | 44,860 | 0.243 | 119.89 | 12.7 | 36.23 | 33,624 | GCA_013358455 | Arias et al. [(2021)](https://www.zotero.org/google-docs/?av9M4U) |
| §Leitch et al. [(2019)](https://www.zotero.org/google-docs/?XcVl9Z) | | | | | | | | | | | | | |

**Supplementary Table 2 References**

[Arias, T., Riaño‐Pachón, D. M., and Di Stilio, V. S. (2021). Genomic and transcriptomic resources for candidate gene discovery in the Ranunculids. *Appl. Plant Sci.* 9. doi: 10.1002/aps3.11407.](https://www.zotero.org/google-docs/?WvAnDI)

[Bartaula, R., Melo, A. T. O., Kingan, S., Jin, Y., and Hale, I. (2019). Mapping non-host resistance to the stem rust pathogen in an interspecific barberry hybrid. *BMC Plant Biol.* 19, 319. doi: 10.1186/s12870-019-1893-9.](https://www.zotero.org/google-docs/?WvAnDI)

[Becker, A., Yamada, Y., and Sato, F. (2023). California poppy (*Eschscholzia californica*), the Papaveraceae golden girl model organism for evodevo and specialized metabolism. *Front. Plant Sci.* 14, 1084358. doi: 10.3389/fpls.2023.1084358.](https://www.zotero.org/google-docs/?WvAnDI)

[Catania, T., Li, Y., Winzer, T., Harvey, D., Meade, F., Caridi, A., et al. (2022). A functionally conserved STORR gene fusion in *Papaver* species that diverged 16.8 million years ago. *Nat. Commun.* 13, 3150. doi: 10.1038/s41467-022-30856-w.](https://www.zotero.org/google-docs/?WvAnDI)

[Filiault, D. L., Ballerini, E. S., Mandáková, T., Aköz, G., Derieg, N. J., Schmutz, J., et al. (2018). The *Aquilegia* genome provides insight into adaptive radiation and reveals an extraordinarily polymorphic chromosome with a unique history. *eLife* 7, e36426. doi: 10.7554/eLife.36426.](https://www.zotero.org/google-docs/?WvAnDI)

[Guo, L., Winzer, T., Yang, X., Li, Y., Ning, Z., He, Z., et al. (2018). The opium poppy genome and morphinan production. *Science* 362, 343–347. doi: 10.1126/science.aat4096.](https://www.zotero.org/google-docs/?WvAnDI)

[Hori, K., Yamada, Y., Purwanto, R., Minakuchi, Y., Toyoda, A., Hirakawa, H., et al. (2018). Mining of the Uncharacterized Cytochrome P450 Genes Involved in Alkaloid Biosynthesis in California Poppy Using a Draft Genome Sequence. *Plant Cell Physiol.* 59, 222–233. doi: 10.1093/pcp/pcx210.](https://www.zotero.org/google-docs/?WvAnDI)

[Huang, H., Liang, J., Tan, Q., Ou, L., Li, X., Zhong, C., et al. (2021). Insights into triterpene synthesis and unsaturated fatty-acid accumulation provided by chromosomal-level genome analysis of *Akebia trifoliata* subsp. *australis*. *Hortic. Res.* 8, 33. doi: 10.1038/s41438-020-00458-y.](https://www.zotero.org/google-docs/?WvAnDI)

[Johns, J., Escalona, M., Nguyen, O., Fairbairn, C. W., Beraut, E., Hodges, S., et al. (2022). The reference genome of the Serpentine columbine (*Aquilegia eximia*). Available at: https://www.ncbi.nlm.nih.gov/assembly/GCA_023053565.1.](https://www.zotero.org/google-docs/?WvAnDI)

[Leitch, I. J., Johnston, E., Pellicer, J., Hidalgo, O., and Bennett, M. D. (2019). Angiosperm DNA C-values database.](https://www.zotero.org/google-docs/?WvAnDI)

[Li, Q., Ramasamy, S., Singh, P., Hagel, J. M., Dunemann, S. M., Chen, X., et al. (2020). Gene clustering and copy number variation in alkaloid metabolic pathways of opium poppy. *Nat. Commun.* 11, 1190. doi: 10.1038/s41467-020-15040-2.](https://www.zotero.org/google-docs/?WvAnDI)

[Liu, Y., Wang, B., Shu, S., Li, Z., Song, C., Liu, D., et al. (2021). Analysis of the *Coptis chinensis* genome reveals the diversification of protoberberine-type alkaloids. *Nat. Commun.* 12, 3276. doi: 10.1038/s41467-021-23611-0.](https://www.zotero.org/google-docs/?WvAnDI)

[Pirro, T., and Pirro, S. (2023). The complete genome sequence of *Sanguinaria canadensis*, the Bloodroot. Available at: https://www.ncbi.nlm.nih.gov/assembly/GCA_028566195.1.](https://www.zotero.org/google-docs/?WvAnDI)

[Sun, Y., Deng, T., Zhang, A., Moore, M. J., Landis, J. B., Lin, N., et al. (2020). Genome sequencing of the endangered *Kingdonia uniflora* (Circaeasteraceae, Ranunculales) reveals potential mechanisms of evolutionary specialization. *iScience* 23. doi: 10.1016/j.isci.2020.101124.](https://www.zotero.org/google-docs/?WvAnDI)

[Xie, J., Zhao, H., Li, K., Zhang, R., Jiang, Y., Wang, M., et al. (2020). A chromosome-scale reference genome of *Aquilegia oxysepala* var. *kansuensis*. *Hortic. Res.* 7, 113. doi: 10.1038/s41438-020-0328-y.](https://www.zotero.org/google-docs/?WvAnDI)

[Xu, Z., Li, Z., Ren, F., Gao, R., Wang, Z., Zhang, J., et al. (2022). The genome of *Corydalis* reveals the evolution of benzylisoquinoline alkaloid biosynthesis in Ranunculales. *Plant J.* 111, 217–230. doi: 10.1111/tpj.15788.](https://www.zotero.org/google-docs/?WvAnDI)

[Yang, X., Gao, S., Guo, L., Wang, B., Jia, Y., Zhou, J., et al. (2021). Three chromosome-scale *Papaver* genomes reveal punctuated patchwork evolution of the morphinan and noscapine biosynthesis pathway. *Nat. Commun.* 12, 6030. doi: 10.1038/s41467-021-26330-8.](https://www.zotero.org/google-docs/?WvAnDI)

[Zhang, R.-G., Lu, C., Li, G.-Y., Lv, J., Wang, L., Wang, Z.-X., et al. (2023). Subgenome-aware analyses suggest a reticulate allopolyploidization origin in three Papaver genomes. *Nat. Commun.* 14, 2204. doi: 10.1038/s41467-023-37939-2.](https://www.zotero.org/google-docs/?WvAnDI)

**Supplementary Figure 1. ASTRAL species tree topologies resulting from collapsing IQ-TREE2 gene trees under various bootstrap thresholds**. BS stands for bootstrap threshold. Inferred species trees share the same topology, while BS values vary both for the backbone and the tips.


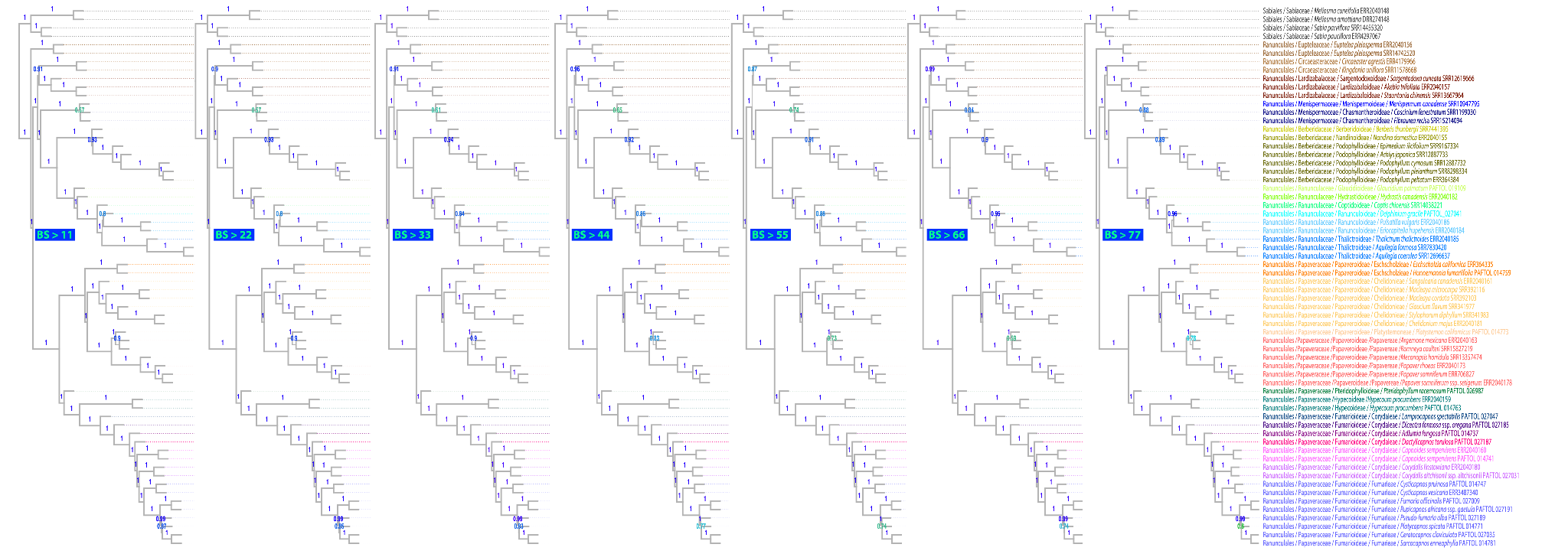

Supplement: Supplementary file 1 [file DataSheet_1.docx]
